# Extracellular Calcium Receptor as a Target for Glutathione and Its Derivatives

**DOI:** 10.3390/ijms23020717

**Published:** 2022-01-10

**Authors:** Thomas Goralski, Jeffrey L. Ram

**Affiliations:** 1 Department of Physiology, Wayne State University, Detroit, MI 48201, USA; Thomas.Goralski@vai.edu; 2 Van Andel Institute, Grand Rapids, MI 49503, USA

**Keywords:** calcium-sensing receptor, L-cysteine-glutathione disulfide, glutathione, ligand-binding, oxidized glutathione, receptor-binding

## Abstract

Extracellular glutathione (GSH) and oxidized glutathione (GSSG) can modulate the function of the extracellular calcium sensing receptor (CaSR). The CaSR has a binding pocket in the extracellular domain of CaSR large enough to bind either GSH or GSSG, as well as the naturally occurring oxidized derivative L-cysteine glutathione disulfide (CySSG) and the compound cysteinyl glutathione (CysGSH). Modeling the binding energies (ΔG) of CySSG and CysGSH to CaSR reveals that both cysteine derivatives may have greater affinities for CaSR than either GSH or GSSG. GSH, CySSG, and GSSG are found in circulation in mammals and, among the three, CySSG is more affected by HIV/AIDs and aging than either GSH or GSSG. The beta-carbon linkage of cysteine in CysGSH may model a new class of calcimimetics, exemplified by etelcalcetide. Circulating glutathionergic compounds, particularly CySSG, may mediate calcium-regulatory responses via receptor-binding to CaSR in a variety of organs, including parathyroids, kidneys, and bones. Receptor-mediated actions of glutathionergics may thus complement their roles in redox regulation and detoxification. The glutathionergic binding site(s) on CaSR are suggested to be a target for development of drugs that can be used in treating kidney and other diseases whose mechanisms involve CaSR dysregulation.

## 1. Introduction

Associations of calcium (Ca) and glutathione in various organs are not well understood. However, molecular modeling by Wang et al. [1] identified a glutathione binding site on the extracellular calcium sensing receptor (CaSR) and demonstrated glutathione-elicited changes in extracellular calcium responses mediated by CaSR transfected into a model cell system. With the perspective of more recent crystallographic studies of CaSR [2,3,4,5] and docking studies of various glutathionergic ligands with CaSR, this paper explores the potential actions that circulating glutathionergic compounds (reduced glutathione (GSH, PubChem CID 124886); oxidized glutathione (GSSG, PubChem CID 65359); and another circulating oxidized derivative of glutathione, L-cysteine-glutathione disulfide (CySSG, PubChem CID 10455148) may have that could be mediated via binding to the extracellular domain of CaSR. We propose that CaSR may be an important nexus for the interaction of glutathione and Ca and that the lesser known of these three compounds, CySSG, may have physiological roles in regulating CaSR activity through its receptor-binding activity.

### 1.1. GSH Synthesis and Relationship to GSH-Derivatives

GSH is a tripeptide (gamma-glutamyl-cysteinyl-glycine) made by non-ribosomal mechanisms. The rate-limiting enzyme in GSH synthesis is glutamate-cysteine ligase (GCL, EC 6.3.2.2), whose activity is limited by the amount of enzyme and the availability of cysteine. Glutathione synthase (GSS) couples glycine to the resultant gamma-glu-cys to make GSH. GSH reacts with oxidants via glutathione peroxidase (GPx) to form GSSG, form GS- adducts with various electrophiles (these are often toxicants that are thereby detoxified) via glustathione-S-transferases (GSTs), or glutathionylates proteins through reactions mediated by glutaredoxins and thioredoxins. Alternatively, GSH is transported out of cells where it can undergo further reactions. GSH can be resynthesized from GSSG by the actions of glutathione disulfide reductase (GR). 

**Figure 1:**
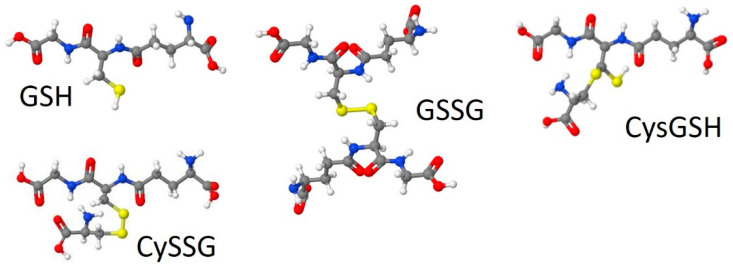
Structures of glutathione (GSH), L-cysteine-glutathione disulfide (CySSG), glutathione disulfide (oxidized glutathione, GSSG), and cysteinyl glutathione (CysGSH). Models were drawn by Jmol, through an interface on Wikipedia (GSSG) or at http://biomodel.uah.es/en/DIY/JSME/draw.en.htm (GSH, CySSG, and CysGSH; accessed 10 and 11 August 2021), based on CID structures 124886 for GSH, 10455148 for CySSG, 65359 for GSSG, and 3080690 for CysGSH, from Pub-Chem (URL: https://pubchem.ncbi.nlm.nih.gov, accessed 22 August 2021).

CySSG can be formed intracellularly or extracellularly by thiol-disulfide exchange with cystine [6], either spontaneously or via enzymatic catalysis by a thioltransferase [7,8]. CySSG is also produced spontaneously by the reaction of GSSG and cysteine [6]; gamma-glutamyltransferase (GGT) produces CySSG and cystine from GSSG, a reaction that can be inhibited by AT-125 (Acivicin) [9]. GGT also degrades CySSG, which can also be inhibited by Acivicin [9]. All three forms of glutathione are found in mammalian circulation [10,11,12,13,14,15]. Structures of the three circulating forms of glutathione (GSH, GSSG, and CySSG) are shown in Figure 1. A fourth structure in Figure 1, designated as cysteinyl glutathione (CysGSH), has cysteine coupled to glutathione through a covalent linkage to the beta carbon of the cysteine in the glutathione backbone and retains the hydrogen atom on one of the sulfurs the two cysteines in the structure, has also been described (PubChem CID 3080690).

**Figure 2:**
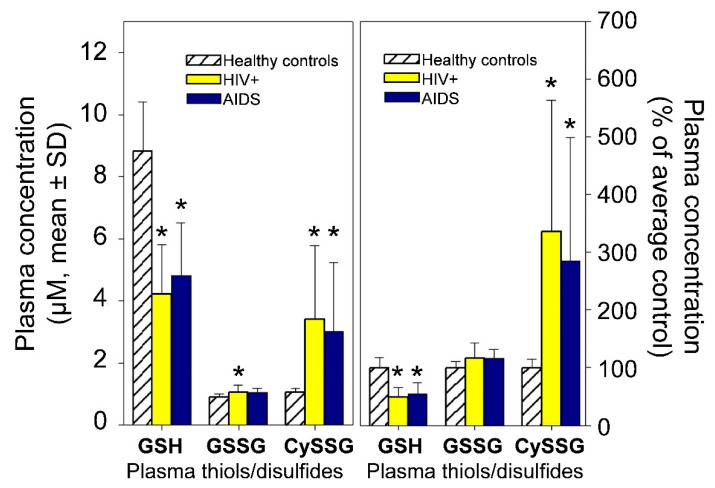
Plasma GSH, GSSG, and CySSG in 10 healthy (HIV-negative) people, 30 HIV-positive, and 23 AIDS patients. (**Left**) Derived from Figure 1 and the text of Walmsley et al. [15]. (**Right**) the same data plotted as per cent of average healthy controls. * *p* < 0.05 compared to controls.

Plasma GSH and CySSG are usually in the 1–10 µM range (e.g., Figure 2, derived from data in Walmsley et al. [15]), while GSSG is often 1 µM or less [10,15]. Many studies have used enzymatic methods to measure GSH; “oxidized glutathione” is often also measured after reduction of oxidized forms to GSH; however, this method does not distinguish between the two oxidized forms of glutathione. Measurement of both oxidized forms of glutathione requires HPLC, which can distinguish between CySSG and GSSG. In healthy people over a broad age range, plasma levels of GSH and CySSG are correlated (Pearson correlation coefficient = 0.622, *p* < 0.001 [10]). Plasma redox pairs have different Eh values (GSH/GSSG, −140 mV; Cys/CySS, −72 mV; and Cys-GSH/CySSG, −110 mV; [10]), indicating that relative concentrations of the reactants have different chemical potentials and are not at redox equilibrium in circulation. CySSG is also found in non-mammalian species, most notably in the polychaete *Nereis succinea* where it functions as a spawning pheromone [16,17,18,19].

### 1.2. CaSR Function in Parathyroid, Kidney, and Other Tissues

CaSR was first identified in parathyroid gland, where it solved the long-standing problem of what receptor mediated up- and down-regulation of parathyroid hormone (PTH) synthesis and release by Ca [20]. Unlike most secretory processes, PTH release is decreased by increases of extracellular Ca. CaSR was discovered to be a G-protein-coupled receptor that mediates responses in parathyroid cells by activating Gq, which regulates phospholipase C (PLC), and Gi, which inhibits cAMP synthesis (as reviewed by Ward [21]). CaSR activity is affected by small changes in Ca in the physiological extracellular range (1–10 mM) [20], and its sensitivity can be shifted by various agents, such as amino acids [22]. 

CaSR is found in many other tissues. In kidney, CaSR expression is particularly high in thick ascending limb (TAL) [21] but also occurs in many renal tissues, including proximal tubule, collecting duct, and juxtaglomerular apparatus [23]. Although approximately 65% of filtered Ca is reabsorbed in the proximal tubule, the proximal reabsorption is mostly not subject to regulatory control. Ca reabsorption in TAL and distal convoluted tubule is regulated in part by CaSR, coupled via G-protein mechanisms to cellular responses. The effect of these actions is to decrease cAMP, which would inhibit the luminal membrane cAMP-dependent Na-K-Cl cotransporter [24,25,26], thereby decreasing Na-reabsorption and inhibiting luminal (apical) K channels via phospholipase A2 and P-450 mediated synthesis of 20-hydroxyeicosatetraenoic acid (20-HETE) [27,28]. These multiple actions of the TAL CaSR cause changes in the transluminal voltage that ultimately cause a decrease in paracellular Ca reabsorption. Numerous other actions in kidney mediated by CaSR include increases in TAL PGE2 production [29], changes in aquaporin trafficking and water transport regulation in renal collecting duct [30], decreases in renin secretion by juxtaglomerular cells [31] and stimulation of claudin-14 expression in TAL mediated by a microRNA-signaling pathway downstream from CaSR activation [32]. 

CaSR protein is also expressed in the gastrointestinal system, bone cells, the nervous system, etc., where these receptors may mediate other Ca-sensitive responses [33]. Among other tissues expressing CaSR are liver cells that stimulate bile flow [34], endothelial cells and vascular smooth muscle cells in many tissues [35] notably in pulmonary arteries [36], pancreatic beta cells [37,38], and taste buds [39,40,41].

### 1.3. Amino Acid and Peptide Modulation of CaSR Activity

The sensitivity of the CaSR to Ca is enhanced by a variety of naturally occurring organic molecules of which amino acids were among the earliest to be described; tryptophan, phenylalanine, tyrosine, and histidine are among the most effective modulators of CaSR activity, generally having EC50 concentrations in the range of 1–10 mM [42,43,44]. Zhang et al. [5] discovered a novel derivative of tryptophan, L-1,2,3,4-tetrahydronorharman-3-carboxylic acid, bound to CaSR and having EC50 of approximately 2 µM. In studies of CaSR in taste buds, modulatory effects of small gamma-glutamyl peptides have been the focus, among which the most effective dipeptides were gamma-glutamyl-alanine, gamma-glutamyl valine, and gamma-glutamyl-cysteine [39,40].

Larger peptides have also been shown to modulate the activity of CaSR, including GSH and GSSG at micromolar and lower concentrations. The study of gamma-glutamyl peptides found that CaSR activity was enhanced by GSH as well as other gamma-glutamyl tripeptides, including gamma-glutamyl-S-methylcysteinylglycine and gamma-glutamylvalylglycine [39,40], generally exhibiting EC50 values in the micromolar range. Wang et al. [1], studying HEK-293 cells transfected with CaSR and tested in a calcium release assay, showed that the Ca response was enhanced in the presence of either GSH or GSSG. EC50 values were <1 µM for both, compared to an EC50 for phenylalanine of 300 µM in the same assays [1]. Neither CySSG nor CysG were tested.

Of particular note is that both GSH and GSSG produced similar physiological responses, supporting the idea that these responses are mediated by binding to a receptor and are not due to interactions with intracellular redox mechanisms, which might have been an alternative explanation if only one of them had been active. A similar conclusion that the action of glutathione is associated with binding, not redox regulation, has been drawn regarding the male spawning response of the polychaete *Nereis succinea*, which is also equally well activated by GSH and GSSG [19]. In the case of *N. succinea*, however, CySSG has also been tested and is effective at eliciting the response at about ten times lower concentration than either GSH or GSSG [19]. Unfortunately, little is known about the structure of the glutathionergic receptor in *N. succinea* and whether it may be part of the C-family of G-protein coupled receptors from which CaSR evolved in vertebrates. Nevertheless, the binding sites of all three of these glutathionergic compounds to potential receptors is of interest.

## 2. CaSR Structure, including Ca, Calcimimetic, Calcilytic, and Peptide Binding Sites

**Figure 3:**
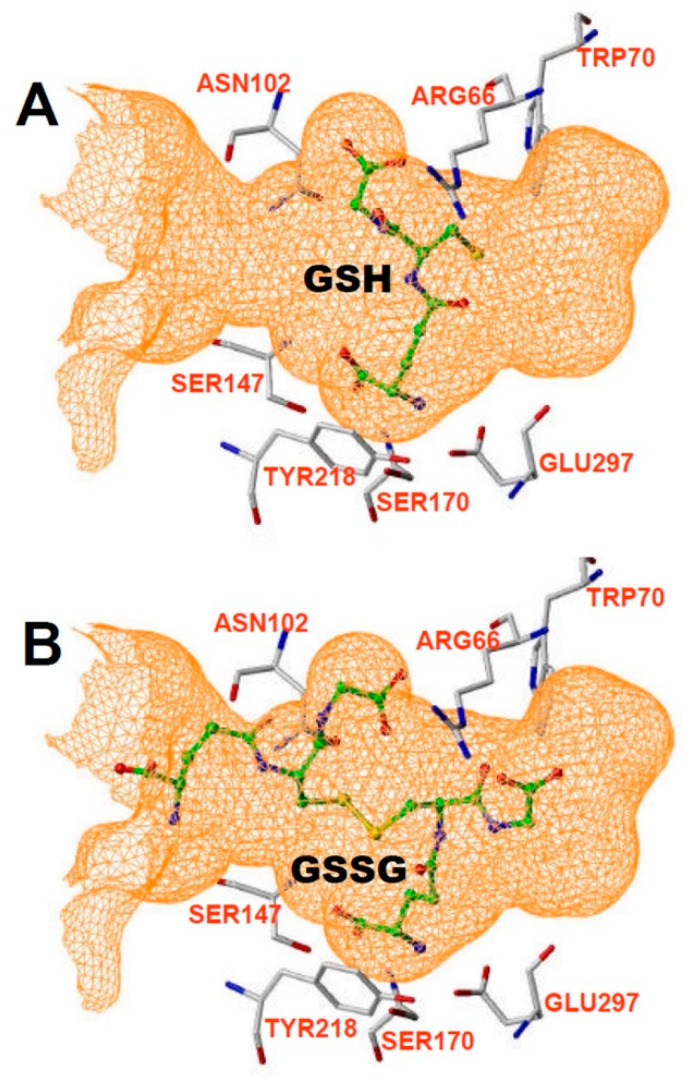
Binding pocket from Wang et al. [1], showing that it can accommodate either (**A**) GSH or (**B**) GSSG.

CaSR is part of the C class of G-protein coupled receptors (GPCRs). The structure has been described as a snake (transmembrane domain, TMD) and a Venus fly-trap (extracellular domain, ECD). Ca binds to the ECD, and several sites on the ECD have been suggested as critical active binding locations for metal ion activation of CaSR [4,5,45]. The sensitivity of CaSR to Ca can be affected by agents that bind to the TMD, including the clinically approved calcium “mimetic” agent Evocalcet [46] and others [47,48,49]. Allosteric antagonists targeting the TMD include NPS 2143 and others [50,51,52]. Calcimimetics that target the ECD have also been developed, including Parsabiv (etelcalcitide, [53,54]. The binding site for amino acids to CaSR is also located in the ECD, at a location consistent with the general structure of the receptor as part of the C family of GPCRs, which includes amino acid binding members that mediate metabotropic responses to glutamate and glycine (reviewed by [1]). By in silico homology modeling of the binding site and subsequent functional studies, Wang et al. [1] determined that the amino acid binding site of CaSR was larger than in the other family C receptors, and that both GSH and GSSG can readily fit in the canonical amino acid binding site (Figure 3, reproduced from [1]).

More recent crystallographic and cryo-EM studies of CaSR have provided direct evidence for the binding pocket(s) of amino acids and larger molecules in the ECD binding pocket of the receptor [3,4,55]. Zhang et al. [5] studied the binding of tryptophan and an unexpected tryptophan derivative bound in crystalized hCaSR ECD. Tryptophan and its derivative bound in a binding pocket that was surrounded by many of the same amino acid residues predicted by Wang et al. [1], including W70, T145, S147, and S170, Y218, and E297. A similar set of residues adjacent to the binding pocket were also determined by Ling et al. 2021 using cryo-EM. The binding pocket described by Zhang et al. [5] was noted to be significantly larger than that of mGluR1, supporting the idea that CaSR could bind much larger ligands than the amino acids it has thus far been crystalized with. In further support of this idea, Zhang et al. [5] also commented that besides binding the somewhat larger tryptophan derivative, the binding site also appears to contain a bicarbonate molecule.

**Figure 4:**
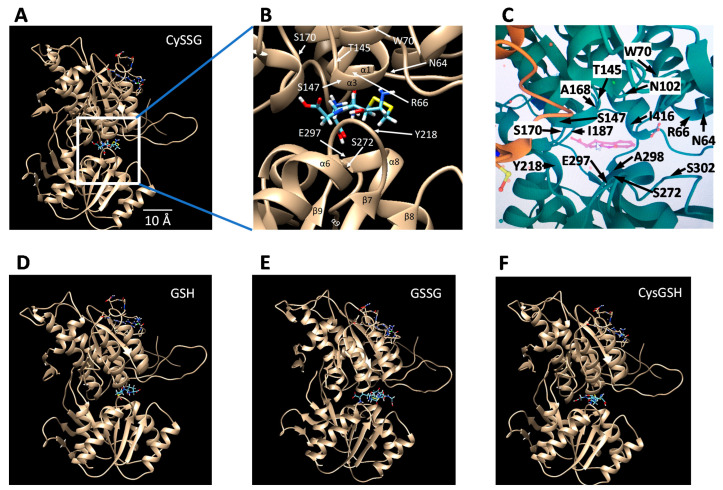
Potential docking sites of glutathionergics to the ECD of CaSR determined with the SwissDock docking simulator [56], and visualized via UCSF Chimera [57] visualization software. Images show Chain B of the CaSR ECD structure with ligands (**A**) and (**B**) CySSG, (**D**) GSH, (**E**) GSSG, and (**F**) CysGSH, docked in the binding site corresponding to where tryptophan and (**C**) a tryptophan derivative [5] are known to bind. Docking simulations utilized the pdb ID 5FBK 3d structure of the CaSR ECD. CySSG and CysGSH were obtained from PubChem CID 44119801 and CID 3080690, respectively, converted from 3d .XML files to .Mol2 files using Open Babel, version 2.3.1, http://openbabel.org (accessed 28 August 2021), ref. [58]; GSH was obtained from Zinc ID 3830891 as a .Mol2 file; GSSG was obtained from zinc ID 3870129 as a .Mol2 file. Pdb ID 5FBK was modified to contain only Chain B of the receptor. The docking region of interest in Chain B was restricted to SwissDock’s XYZ center—35, 17, and 27 with XYZ sizes of 15. (**B**) zooms into the binding site of (**A**) to identify the locations of CaSR residues adjacent to CySSG, for comparison to (**C**) the comparable region identified and labeled as in Zhang et al. [5] and rendered here from the RCSB PDB (http://rcsb.org, accessed on 28 August 2021, ref. [59]) of PDB ID of 5FBH [5] (same as 5FBK but with methylcyclotryptophan included (pink molecule in the center). The yellow side chain of an amino acid in the lower left of (**C**) is part of C482 of the A chain. Identification of alpha helices and beta sheet regions and the numbering and location of the residues in (**B**) is derived from data given by Zhang et al. [5], Figures 1 and S1. The length calibration in (**A**) is based on the turn-to-turn distance of the illustrated alpha helices (5.4 Å).

The ECD binding pocket based on these recent structural studies is illustrated in this paper in Figure 4, showing that it can also easily accommodate CySSG (Figure 4A,B), in a similar location to the position of Zhang et al.’s tryptophan derivative [5] (illustrated in Figure 4C). In the illustrated configuration of CySSG, the calculated change in Gibbs free energy of binding (∆G) is −9.43 Kcal/Mol for CySSG, as illustrated using SwissDock [56] and UCSF Chimera [57]. In a comparable configuration the calculated GSH ∆G is only −6.54 Kcal/Mol (Figure 4D). 

The average calculated free energy of binding to CaSR of CySSG, GSH, GSSG, and CysGSH, as determined by SwissDock [56], helps estimate the likely relative affinity of the ligands for the receptor. SwissDock analysis of approximately 250 docking positions was −7.22 ± −0.16 (mean ± SD) Kcal/Mol for CySSG, −6.60 ± 0.15 Kcal/Mol for GSH, and −6.54 ± 0.17 Kcal/Mol for GSSG, and −7.19 ± −0.07 for CysGSH (*n* = 4 replications each; 1-way ANOVA, *p* < 0.0001, ANOVA; Tukey HSD post-hoc tests: CySSG v. GSH, *p* = 0.0001; CySSG v. GSSG, *p* = 0.0003; CysGSH v. GSH, *p* = 0.0004; and CysGSH v. GSSG, *p* = 0.0004; all other comparisons, *p* > 0.5). Among the naturally occurring potential ligands, these estimates of the Gibbs free energy of binding indicate a greater average affinity of CaSR for CySSG than for either GSH or GSSG, and therefore greater binding of CySSG at comparable temperature and concentrations, assuming no additional steric effects related to accessibility of the binding site. The docking behavior on CaSR of CysGSH is similar to CySSG.

## 3. Associations of Ca with Glutathionergic Metabolism

Several experiments have examined the association of Ca with GSH metabolism. The rise in GSH synthesis in RAW264.7 macrophage tumor cells in response to gamma rays is Ca dependent [60]: GCLC mRNA increased with a similar time course to GSH, a response that was inhibited in cells cultured with 1 mM EGTA (Ca chelator) or BAPTA/AM (intracellular Ca chelator). Coordinate regulation of gene transcription for GCLC, GSS, and other glutathione-regulating genes is mediated by Nrf2, an activator of antioxidant response elements in their 5′-flanking promoter regions [61,62]. In human keratinocytes, activation of Nrf2 by arsenite was reduced by depleting cells of Ca in Ca-free media [63]. Ca-calmodulin inhibition of CK2 kinase activity mediates the response, i.e., low Ca results in higher CK2 activity, which phosphorylates Nrf2, making it more vulnerable to degradation. A treatment that reduced UV-radiation damage in lens tissue decreased the expression of CaSR at the same time that markers of oxidative stress (SOD and “T-AOC,” said to measure total antioxidant content, but is not a direct measurement of glutathione) were increased [64].

Changes in glutathione metabolism in liver cells are particularly significant as the liver is the major source of circulating glutathione [65]. GSH in hepatocytes was increased by 3.5 mM extracellular Ca compared to 0 mM extracellular Ca [66]. In a recent study, ionizing radiation increased liver Ca, accompanied by large decreases in total glutathione and glutathione-regulating enzymes, interpreted as a large relative increase in oxidant status, as compared to antioxidant status [67]. 

These previous studies relating Ca and glutathione have generally interpreted their findings in terms of redox status of cells and, except for the study UV-radiation damage in lens tissue [64], have not considered possible roles of CaSR in the mechanisms that might be involved. Nevertheless, evidence exists to indicate that glutathione metabolism may be interactive with calcium signaling and possibly related to extracellular calcium concentration via CaSR.

## 4. Proposed Role of Glutathionergics in Regulating CaSR Function

The above information can be summarized as follows: First, GSH and its oxidized derivatives CySSG and GSSG are found in mammalian circulation at micromolar concentrations and exhibit changes correlated with age and health. Second, CaSR is found in many tissues, including parathyroid gland, kidney, and bone, where it participates in regulation and utilization of extracellular Ca. Third, amino acids and peptides, including GSH and its derivatives, can sensitize CaSR responses to Ca; in taste buds GSH and related compounds can activate CaSR under ambient Ca conditions. Fourth, consistent with the functional effects of glutathionergics on CaSR, the binding pocket at which amino acids exert their effects on CaSR is large enough to accommodate peptides, including glutathione and its oxidized derivatives. Fifth, extracellular Ca in the same concentration range as is regulated by CaSR in parathyroid, kidney, and bone tissues can modify glutathione synthesis, particularly in hepatocytes, the major source of circulating glutathione. 

Given the above observations, we therefore propose that circulating glutathionergics (GSH, GSSG, and/or CySSG) bind to and sensitize CaSR to extracellular Ca and thereby participate in the homeostatic regulation of extracellular Ca. A subsidiary hypothesis, based on the physiological principle that homeostatic systems usually have feedback to the source of the regulatory signal, is that extracellular Ca effects on glutathione metabolism in the liver, the major source of circulating glutathionergics, may constitute a feedback mechanism for this hypothesized glutathionergic Ca regulatory mechanism. The association of CySSG changes with age and health and the greater affinity of CySSG for CaSR in model docking simulations may indicate an importance for this derivative of CySSG that has heretofore been overlooked. 

This proposed role broadens our views about the functions of glutathione, emphasizing an extracellular receptor-mediated role for glutathionergics, complementary to their well-known intracellular actions regulating the intracellular redox state of cells. This paper highlights the potential biological actions of plasma CySSG and further emphasizes the peptide binding site on CaSR as a potential target the development of drugs that can be used in treating kidney, Parkinson’s and other diseases.

## 5. Medical Implications

In the period 2015–2018, 37 million Americans had chronic kidney disease (CKD), including 38% of patients age 65 and older [68]. Together, CKD and End-Stage Renal Disease (ESRD) had an annual Medicare cost (2017) of over $120 billion [69]. ESRD is often accompanied by secondary parathyroidism and abnormal Ca homeostasis [70]. Calcimimetics targeting CaSR were developed to enhance the affinity of CaSR for Ca and thereby reduce parathyroid hormone at more modest Ca concentrations [71]. These compounds (e.g., AMG073 (Cinacalcet, Sensipar), FDA approved in 2004 and etelcalcitide (Parsabiv), approved in 2017) are effective but expensive (about $10,000 per patient per year [72]) and may be accompanied by side effects [73]. Another renal condition associated with abnormal CaSR function includes kidney stones [74].

While the earliest calcimimetics, such as Cinacalcet targeted the TMD of CaSR; the more recent category of calcimimetics represented by etelcalcetide apparently produce their response by binding at a site near the amino acid/peptide binding site of the ECD [2,54] Alexander, Hunter, Walter, Dong, Maclean, Baruch, Subramanian and Tomlinson [54] suggested that etelcalcetide’s cysteine residue may undergo a critical disulfide interaction with CaSR ECD cysteine residue C482, which is supported by cryoelectronmicroscopy structural analysis [2] of CaSR showing elelcalcitide bound by disulfide bonds to C482 in the homodimer interface CaSR. The nearby position of C482 to the amino acid/peptide binding pocket of CaSR is illustrated in this paper in Figure 4C, in which C482 in the A chain of CaSR is shown as the yellow residue in the lower left part of the figure. A comparably near C482 in the B chain is also present but obscured in Figure 4C behind other amino acids. 

It has not escaped our attention that etelcalcetide, like the naturally occurring CySSG, has a critical cysteine residue connected to its peptide backbone structure. Interestingly, CysGSH, the other cysteine-glutathione compound that we have highlighted in this review, is linked by a covalent bond between the cysteine S and one of the backbone carbons, similar to the linkage of cysteine to the peptide backbone of etelcalcetide, as illustrated by Alexander, Hunter, Walter, Dong, Maclean, Baruch, Subramanian and Tomlinson [2,54]. Whether CySSG or CysGSH may bind in the location demonstrated for etelcalcitide is a matter for future modeling and empirical analysis.

According to the perspective presented in this paper, etelcalcetide may, in this respect, be mimicking the functional actions of a naturally circulating glutathionergic compound, CySSG. The amino acid sensitizers of CaSR are active at mM concentrations. In contrast, glutathionergics bind to and sensitize the receptor at µM concentrations. Like etelcalcetide, glutathionergics may be the basis for developing lead compounds for a “next generation” of CaSR sensitizers, especially if it can be determined that CySSG is the most effective of the glutathionergics at producing physiological responses. In this regard, CysGSH should also be considered as a possible CaSR sensitizer.

The actions of glutathionergics on CaSR may also have implications for other functions besides Ca homeostasis. Aside from possible roles in renal disease, changes in glutathionergics and CaSR function may also have roles pathology in HIV-AIDs, osteoporosis, pulmonary hypertension, and neurodegenerative diseases. HIV/AIDS is accompanied hypercalciuria and osteopenia [75,76] and, as already reviewed (Figure 2), changes in plasma levels of GSH and its oxidized derivatives also occur in HIV/AIDS [15]. With respect to heart disease, mutations in promotor regions of subunits of glutamate-cysteine ligase (GCL, the rate-limiting enzyme for GSH synthesis) are associated with increased risk for myocardial infarction [77], which could be related to GSH-potentiation of endothelial nitric oxide release [78] and the extracellular calcium receptor (CaSR) in cardiac microvascular endothelial cells [79]. With regard to osteoporosis, osteoclasts and osteoblasts have CaSR (reviewed in [80]), and osteoporosis is associated with changes in glutathione-associated enzymes [81,82]. A proposed mediator for some forms of pulmonary hypertension is CaSR in pulmonary vasculature [83]. Pulmonary hypertension is also accompanied by changes in plasma glutathionergics which have been suggested as targets for treatment [84]. Degenerative diseases of the brain such as Parkinson’s and Alzheimer’s disease may also be affected by both glutathionergics [85,86,87] and CaSR [88,89,90,91] in brain tissue; however, their neural and systemic interactions are complex and beyond the scope of the present review. In almost every case where glutathionergics have been investigated, their hypothesized roles have been interpreted based on their functions in redox regulation or detoxification and not based on a possible CaSR-receptor mediated role that is hypothesized here.

## 6. Conclusions

Three key observations are reviewed in this paper:

1. *Glutathionergic compounds can produce biological responses through receptor binding mechanisms on CaSR.* Most discussions of the roles of GSH and its derivatives focus almost exclusively on their functions in regulating the redox states of cells and in detoxifying chemicals (e.g., Lu [92]). The idea that glutathionergics may also function as ligands that bind to receptors such as CaSR via structures that are not necessarily affected by their redox state broadens the types of roles that they can have. Furthermore, receptor function involves reversible binding, in contrast to covalent detoxification mechanisms.
2. *A role for plasma CySSG*. CySSG is present in plasma at concentrations greater than GSSG and nearly as high as GSH; in some medical conditions (e.g., HIV/AIDS) the ratio of CySSG to GSH changes as much as 5-fold. The higher affinity calculated in this paper for CySSG binding to CaSR, compared to GSH and GSSG, suggests that CySSG may even be the preferred ligand at the peptide binding site on CaSR. However, the presence and possible functions of CySSG have gone largely uncommented upon, even in the publications with the most extensive human plasma measurements of CySSG [10,15], despite CySSG-specific significant changes and correlations in the texts of the papers. CySSG is known to stimulate an important receptor-mediated response in invertebrate reproduction [19]. The proposal here is that CySSG has important functions in mammalian Ca homeostasis.
3. *Glutathionergics may be lead compounds for new regulators of CaSR activity for clinical treatments.* As noted above, the presence of a cysteine residue attached to a peptide backbone, found in CySSG, CysGSH, and etelcalcetide, may point the way to further development of compounds that sensitize or antagonize CaSR through actions in the amino acid/peptide binding pocket of the CaSR ECD. A variety of disease conditions associated with changes in CaSR activity and affected by GSH and its derivatives may be subject to treatment with new drugs derived from this understanding.

## Data Availability

The data underlying the descriptive statistics for ∆G calculations presented in this study are available on request from the corresponding author.
